# Selective Gene Transfer to the Retina Using Intravitreal Ultrasound Irradiation

**DOI:** 10.1155/2012/412752

**Published:** 2012-01-31

**Authors:** Shozo Sonoda, Katsuro Tachibana, Toshifumi Yamashita, Makoto Shirasawa, Hiroto Terasaki, Eisuke Uchino, Ryo Suzuki, Kazuo Maruyama, Taiji Sakamoto

**Affiliations:** ^1^Department of Ophthalmology, Graduate School of Medical and Dental Sciences, Kagoshima University, Kagoshima 890-8520, Japan; ^2^Department of Anatomy, School of Medicine, Fukuoka University, Fukuoka 814-0180, Japan; ^3^Department of Biopharmaceutics, School of Pharmaceutical Sciences, Teikyo University, Sagamihara 229-0195, Japan

## Abstract

This paper aims to evaluate the efficacy of intravitreal ultrasound (US) irradiation for green fluorescent protein (GFP) plasmid transfer into the rabbit retina using a miniature US transducer. Intravitreal US irradiation was performed by a slight modification of the transconjunctival sutureless vitrectomy system utilizing a small probe. After vitrectomy, the US probe was inserted through a scleral incision. A mixture of GFP plasmid (50 *μ*L) and bubble liposomes (BLs; 50 *μ*L) was injected into the vitreous cavity, and US was generated to the retina using a SonoPore 4000. The control group was not exposed to US. After 72 h, the gene-transfer efficiency was quantified by counting the number of GFP-positive cells. The retinas that received plasmid, BL, and US showed a significant increase in the number (average ± SEM) of GFP-positive cells (32 ± 4.9; *n* = 7; *P* < 0.01 ). No GFP-positive cells were observed in the control eyes (*n* = 7). Intravitreal retinal US irradiation can transfer the GFP plasmid into the retina without causing any apparent damage. This procedure could be used to transfer genes and drugs directly to the retina and therefore has potential therapeutic value.

## 1. Introduction

Ultrasound (US) increases the permeability of the plasma membrane and reduces the thickness of the unstirred layer at the cell surface, thereby facilitating the entry of DNA into cells. Furthermore, a combination of low-intensity US and microbubble (MB) echocontrast agents allows direct DNA transfer into the cytosol through small pores in the cells caused by cavitation effects, and dramatically enhances gene transfection both *in vitro* and *in vivo* [1–4]. Previously, our group reported that combination of US and MB increases the induction efficiency of plasmid DNA in the surface of ocular tissues such as cornea, conjunctiva, and eyelid [1, 5, 6]. The retina deeper part of ocular tissue was more hard to deliver DNA because of difficulties of US exposure, we also demonstrated a possibility of transcorneal US irradiation with MB transfer of DNA plasmids into the retina (Sonoda S, et al. IOVS 2006;47:ARVO E-Abstract 828). However, the efficiency of DNA plasmid induction was not so high and the applications of US irradiation to retina were limited to external irradiation form the cornea due to US probe size. Furthermore the lack of targeting ability of this transcorneal method meant that unpredictable effects might occur in other tissues, such as the lens, iris, and ciliary body. Selective retinal transfection would thus be advantageous to improve induction efficiency and avoid unexpected US exposure.

A clinical application of a new therapeutic US method for treating thrombosis has been developed [7, 8]. This method employs a miniature US transducer at the tip of a MicroLysUS infusion catheter (EKOS Corp., Bothell, USA), which approaches the target site via arterial vessels, and has been shown to improve clinical outcomes [7–9]. We have explored the use of this concept to apply US at shorter distances with a smaller probe, which should allow us to irradiate US selectively and to minimise the damage to the other ocular tissues. Our group developed a tiny US probe as small as a 19-gauge needle which can insert to vitreous cavity and exposure US selectively to retina. The aim of the present study was to evaluate the plasmid DNA deliver efficacy of intravitreal US irradiation using a miniature US transducer. This manuscript is the first attempt of intravitreal US irradiation to retina.

## 2. Methods

**Figure 1:**
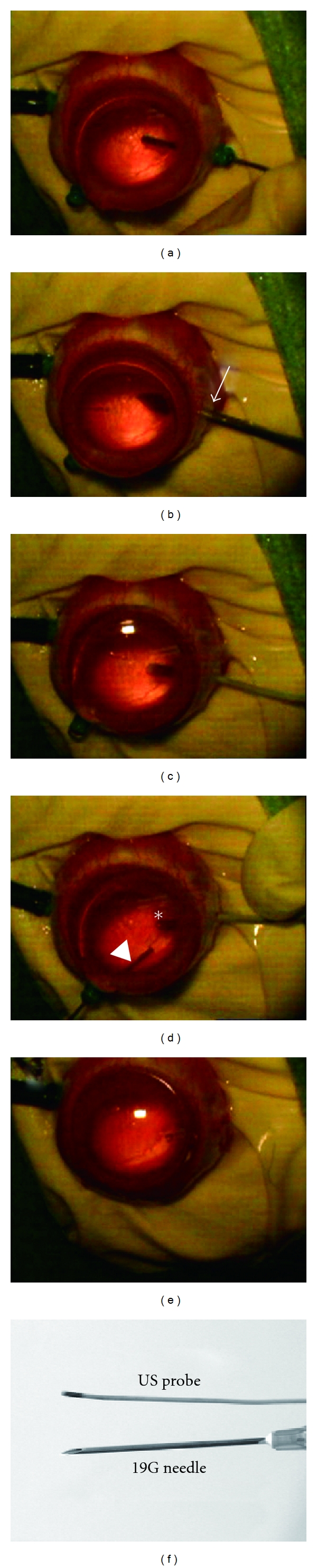
(a) Vitrectomy was performed with a 25-gauge vitrectomy system with rabbit eye. (b) Enlarge the superonasal incision with 19G needle (arrow) for the insertion of the US probe. (c) The eye ball had preserved intact after insertion of the US probe. (d) Bubble liposome was injected through the superotemporal port (arrowhead) and US irradiation (asterisk) was performed simultaneously. (e) The eye ball was kept intact after whole procedures. (f) The US probe size is as small as a 19G needle.

All of the animals were handled humanely in strict accordance with the Association for Research in Vision and Ophthalmology (ARVO) Statement for the Use of Animals in Ophthalmic and Vision Research, and with the approval of the ethics board of Kagoshima University, Japan. Male New Zealand albino rabbits (age = 14 weeks; body weight = 3 kg; KBT Oriental Co. Ltd., Saga, Japan) were anesthetised with an intramuscular injection of ketamine hydrochloride (14 mg/kg) and then xylazine hydrochloride (14 mg/kg). The procedures specifically followed the transconjunctival sutureless vitrectomy system (TSV) [10, 11]. Using a trocar cannula (25G trocar cannula system, Alcon, Fort Worth, Texas, USA), three incisions were made in the inferotemporal, superotemporal, and superonasal quadrants, and an infusion cannula was inserted into the inferotemporal incision (Figure 1(a)). Vitrectomy was performed with an accurus 800CS with a 25-gauge TSV (Alcon). The central and preretinal vitreous was excised to allow sufficient room for agent injections. Then, the superonasal incision was enlarged using a 19-gauge needle (Terumo, Tokyo, Japan) to allow the US probe to be inserted (Figures 1(b) and 1(f)).

 A bubble liposome (BL) is a type of MB that has been developed by our group to allow more efficient gene transfer into a target site than conventional MBs [5, 6, 12–14]. The BLs were prepared following the methods described in our previous report [15]. Green fluorescent protein (GFP) coding plasmid (pEGFP-N2, Clontech, Mountain View, CA, USA; 50 *μ*L) was mixed with BLs (50 *μ*L), and then injected at a slow speed (for 30 s) into the generated preretinal space through the cannula, using a syringe with a 27-gauge blunt needle (Nipro, Osaka, Japan). During the injection, the US probe was positioned 1-2 mm from the central part of the optic disc of the retina, and US (frequency = 3 MHz; duty=6%; intensity = 0.15 W/cm^2^; time = 60 s) was applied to the retina using a SonoPore 4000 (NEPA GENE, Chiba, Japan) (Figures 1(c) and 1(d)). The control group was treated using identical procedures but without US exposure (BL + plasmid). Immediately after the US exposure, we confirmed the rupture of the BLs, which indicated the occurrence of cavitation [1, 2, 16]. The sclera incision port, which was used for the US probe, was then sutured with 10-0 nylon (Alcon), and the other microcannulas that did not require sutures were removed (Figure 1(e)). Finally, antibiotic ointment was applied to the conjunctival sac (whole procedure movie; see supplementary file in supplementary material available online at doi:10.1155/2012/412752). We also confirmed the condition used plasmid alone following US irradiation (US + plasmid).

The eyes were enucleated 72 h after treatment, and immediately frozen in liquid nitrogen-cooled isopentane. Serial sections were sliced with a cryostat adjacent area of optic disc where US were exposed. The GFP was observed by laser confocal microscopy (FV-1000; Olympus, Tokyo, Japan). The gene-transfer efficiency was quantified by measuring the intensity of GFP-positive cells randomly selected four sections form each rabbit and calculated average number by three masked observers. Seven rabbit eye were treated for each condition.

## 3. Results

**Figure 2:**
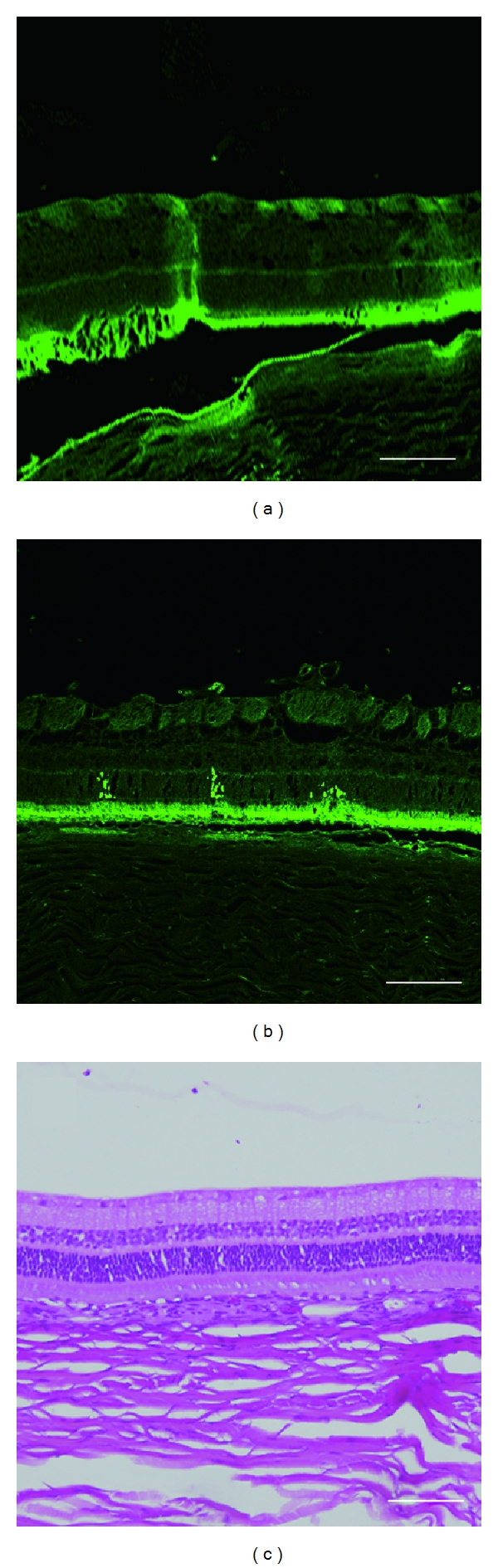
(a) No-GFP positive cells were observed in the control (plasmid + BL without US), (b) In the group treated with plasmid + BL + US, GFP-positive cells were observed exclusively in the area where US was exposed, and located in the outer nuclear layer. (c) H&E staining showed no obvious tissue damage. Bar equals 100 *μ*m.

No GFP-positive cells were observed in the control eyes (*n* = 7; Figure 2(a)); however, the retinas that received plasmid and US concomitantly with or without BL showed GFP-positive cells (Figure 2(b)). Importantly, the GFP-positive cells were limited to the area exposed to US, and were observed mainly in the outer nuclear layer. The average number of GFP-positive cells in BL + plasmid + US group was 32.0 ± 4.9 (mean ± SEM, *n* = 7) per visual field and 4.2 ± 2.7 (mean ± SEM, *n* = 7) in plasmid + US group with ×200 magnification (Table 1). Induction efficiency of BL + plasmid + US was a statistically significant increase (*P* < 0.01, Mann-Whitney *U* test) compared with both control and US + plasmid group.

 Slit-lamp biomicroscopy examinations were performed 1 day and 3 days after treatment, and revealed no obvious tissue damage (i.e., an intact bulb shape, no cells in the anterior chamber, and no preretinal haemorrhage). Histological examinations by hematoxylin and eosin (H&E) staining showed no critical retinal damage in any of the treatment groups (Figure 2(c)).

## 4. Discussion

In this study, direct intravitreal retinal US irradiation transferred the GFP plasmid to the rabbit retina without any apparent tissue damage. Furthermore, combining the GFP plasmid with BLs greatly enhanced the gene delivery. To our knowledge, this is the first report to attempt intravitreal US irradiation with a 25-gauge TSV system. Diameter of US transducers commonly used for gene delivery were 3 mm and more, therefore only transcorneal US irradiation could be available on occasions when US was exposed to the retina, deeper portion of ocular tissue. Our preliminary study using transcorneal US exposure to retina showed (Sonoda S, et al. IOVS 2006;47:ARVO E-Abstract 828), in that study luciferase plasmid was employed instead of GFP, we could not compare accurately, scattered positive staining cells (11.3 ± 3.2, mean ± SEM, *n* = 6) were only observed in superficial layer of retina. In this study with intravitreal US irradiation, the average number of positive staining cells was obviously larger than transcorneal US exposure.

Our group had achieved to minimize the diameter of US probe which is enough small to insert vitreous cavity. On the contrary, the US probe, was not customized for the only purpose for intravitreal US irradiation, still remaining problems are such as ring sonography transducer that generate a 360° circumferential pulse and US intensity was not specially tuned for retina. Even this type of US probe could transfer GFP plasmid into retina. In other word, development of a US probe specialised for ocular tissue, for example, with optimal US intensity and unidirectional US irradiation towards the retina, might increase the gene-transfer efficiency. Furthermore, reducing the size of the US probe could avoid the need for additive sclera incision, reduce the procedure time, and potentiate the no-suture minimal surgery of the 25 G TSV system. Taken together, Intravitreal US irradiation may have potential therapeutic value.

GFP-positive cells were observed exclusively in the area that was exposed to US. This indicates that intravitreal US exposure is more selective for the retina than the transcorneal method, thereby avoiding gene delivery to unexpected areas and minimizing the damage to the surrounding tissues. It is not clear why GFP-positive cells were observed mainly in the outer nuclear layer, although some possible explanations are as follows. In the present procedure, the US intensity was focused on the outer layer of the retina, which might have increased the gene transfer specifically to that layer. Another possibility is that retinal pigment epithelial (RPE) cells that are highly polarised with tight junctions produce a tight blood-retinal barrier [17], and the US intensity used in this study might have not been sufficient to break the RPE barrier. Further experiments will be needed to elucidate these mechanisms.

The present procedure used the BL, which is a type of MB devised by our group that is based on liposome technology, and comprises a polyethylene-glycol-modified liposome (PEG-liposome) containing perfluoropropane (C_3_F_8_)  gas [6, 12, 15]. In the case of intravitreal US, only the combination of BL plus US exposure enhanced the gene-transfer efficiency. As described in previous reports [1, 2, 6], trapped gas inside the BL is cavitated by US, enhanced to form microjet flow, and allows easy gene entry into the cell [13, 15]. The BL has several advantages compared with the conventional and well-studied MB, the Optison (GE Healthcare, Little Chalfont, UK). The BL can be modified to incorporate additional components, such as targeting functions, as it was developed based on liposome technology, besides BL was incompatible with DNA plasmid and drugs easily [18, 19]. Furthermore, components of the BL are already in use in clinical applications, and US has also been used clinically for a long time [15, 20]. Based on these facts, the present method is likely to be safe for human treatment.

Again, the major advantage of this method was selective US irradiation in vitreous cavity with minimal side effect. This concept would be applicable to treat various retinal diseases. Retinal detachment (RD) is one of the good candidate diseases for this method, because the present method can be easily applied during vitrectomy and the gene transfer of neuroprotective or anti-apoptotic factor might prevent the further deterioration of vision [21, 22]. Now we established a rabbit RD model to evaluate the possibility of our method. If this concept would be proven by this model, the application would be widely expanded to such disease as retinitis pigmentosa and glaucoma.

## Supplementary Material

Legend for video: Intravitreal US irradiation was performed with a slight modification of transconjunctival sutureless vitrectomy system utilizing the miniature US probe which was comparable in magnitude to a 19G needle.

## Figures and Tables

**Table 1:** Quantification of gene-transfer efficiency by counting the number of GFP-positive cells.

Condition	Number of GFP positive cell (mean ± SEM)	Mann-Whitney *U* test
control (BL + *plasmid*)	No positive cell	
US + BL + plasmid	32.0 ± 4.9	*P* < 0.01 (versus control and US + plasmid)
US + plasmid	4.2 ± 2.7	*P* < 0.01 (versus control)
